# Risk Factors Associated with Oral Intake Discontinuation in Hospitalized Patients with Aspiration Pneumonia: A Scoping Review

**DOI:** 10.31662/jmaj.2024-0077

**Published:** 2024-12-20

**Authors:** Kokoro Kato, Katharina da Silva Lopes, Emilie Louise Akiko Matsumoto-Takahashi

**Affiliations:** 1 Graduate School of Public Health, St. Luke's International University, Tokyo, Japan; 2 Fujita Health University, Okazaki Medical Center, Aichi, Japan; 3 Institute of Child Nutrition, Federal Research Institute of Nutrition and Food, Karlsruhe, Germany

**Keywords:** Aspiration Pneumonia, patient hospitalized, discontinuation of oral intake, ICHUSHI, scoping review, Japan

## Abstract

**Background:**

Aspiration pneumonia is a prevalent condition, and understanding the risk factors associated with discontinuation of oral intake upon discharge is crucial. This study aimed to identify such factors, thereby providing valuable insights for optimizing the use of limited healthcare resources and enhancing patient and family care.

**Methods:**

In this scoping review, data were collected through ICHUSHI using the search formula “Pneumonia-Aspiration/Thesaurus or Aspiration Pneumonia/All) and (Prognosis/Thesaurus or Prognosis/All).” The inclusion criteria encompassed Japanese patients hospitalized for aspiration pneumonia, with a clear outcome focused on the availability of oral intake. The exclusion criteria included text unavailability, studies from foreign countries, and cases involving not hospitalized patients. The risk of bias for each study was assessed using the Newcastle-Ottawa scale.

**Results:**

Using this search formula, 1,646 articles were initially identified, culminating in the inclusion of six articles for analysis. The investigation revealed five significant risk factors: social status (age and gender), nutritional status (body mass index, Controlling Nutritional Status score, serum albumin, Basal Energy Expenditure, and low body weight), physical swallowing function (ambulatory ability before admission, Food Intake LEVEL scale (FILS), admission origin, bedridden status, Penetration-Aspiration scale, presence of residual pharyngeal material, and Basal Index), pneumonia severity (A-DROP score, a classification tool incorporating age, dehydration, oxygen demand, impaired consciousness, and hypotension), and comorbidities (pneumonia, dementia, mental illness, malignancy, chronic lower respiratory tract involvement, and renal failure).

**Conclusions:**

This scoping review identified five key risk factors associated with oral intake discontinuation upon discharge in patients hospitalized for aspiration pneumonia, providing valuable evidence for future clinical practice.

## Introduction

### Background information

Aspiration pneumonia occurs when a substantial volume of oropharyngeal or upper gastrointestinal tract contents inadvertently passes through the glottis and enters the trachea, resulting in pneumonia ^(1)^. Determining the precise site of pneumonia resulting from aspiration is challenging. Clinically, a diagnosis is established in the presence of lung inflammation and confirmed dysphagia or when aspiration is strongly suspected ^(2)^. Accounting for 5%-15% of community-acquired pneumonia, aspiration pneumonia is associated with factors such as impaired swallowing function, diminished consciousness, gastroesophageal reflux, and reduced cough reflex ^(3)^. Its occurrence spans various settings, including homes, institutions, and hospitals, with prehospitalization medical and nursing environments influencing its onset ^(4), (5)^.

A considerable proportion of patients hospitalized for aspiration pneumonia are older, thereby experiencing a decline in activities of daily living (ADL) posthospitalization due to acute illness ^(6)^. This demographic may also exhibit impaired swallowing function, necessitating food consistency modifications and assistance during post-pneumonia treatment ^(7)^. In some instances, oral intake becomes challenging, thus requiring alternative nutrition. The consequences of these changes may extend to postdischarge living arrangements.

Anticipating the potential for a change in the care setting at discharge compared with prehospitalization could yield numerous benefits for both healthcare providers and patients. This foresight will enable rehabilitation, nursing, and care coordination staff to intervene more efficiently, thereby preventing staff burnout. In addition, it provides the opportunity to inform patients and families beforehand regarding the increased risk of care setting changes, facilitating streamlined decision making, and potentially reducing hospital stays. Aspiration pneumonia is a leading cause of adult mortality in Japan, particularly in the older individuals, and is associated with increased mortality rates with advancing age ^(1)^. Moreover, acute illness can precipitate delirium and disuse, emphasizing the importance of appropriate treatment and prevention.

### Objectives

This study identified risk factors associated with discontinuation of oral intake upon discharge in Japanese patients hospitalized for aspiration pneumonia. The findings aim to provide insights into the efficient utilization of limited healthcare resources and improved patient and family care.

## Materials and Methods

### Study design

The present study is a scoping review.

#### Data collection

**Figure 1.**
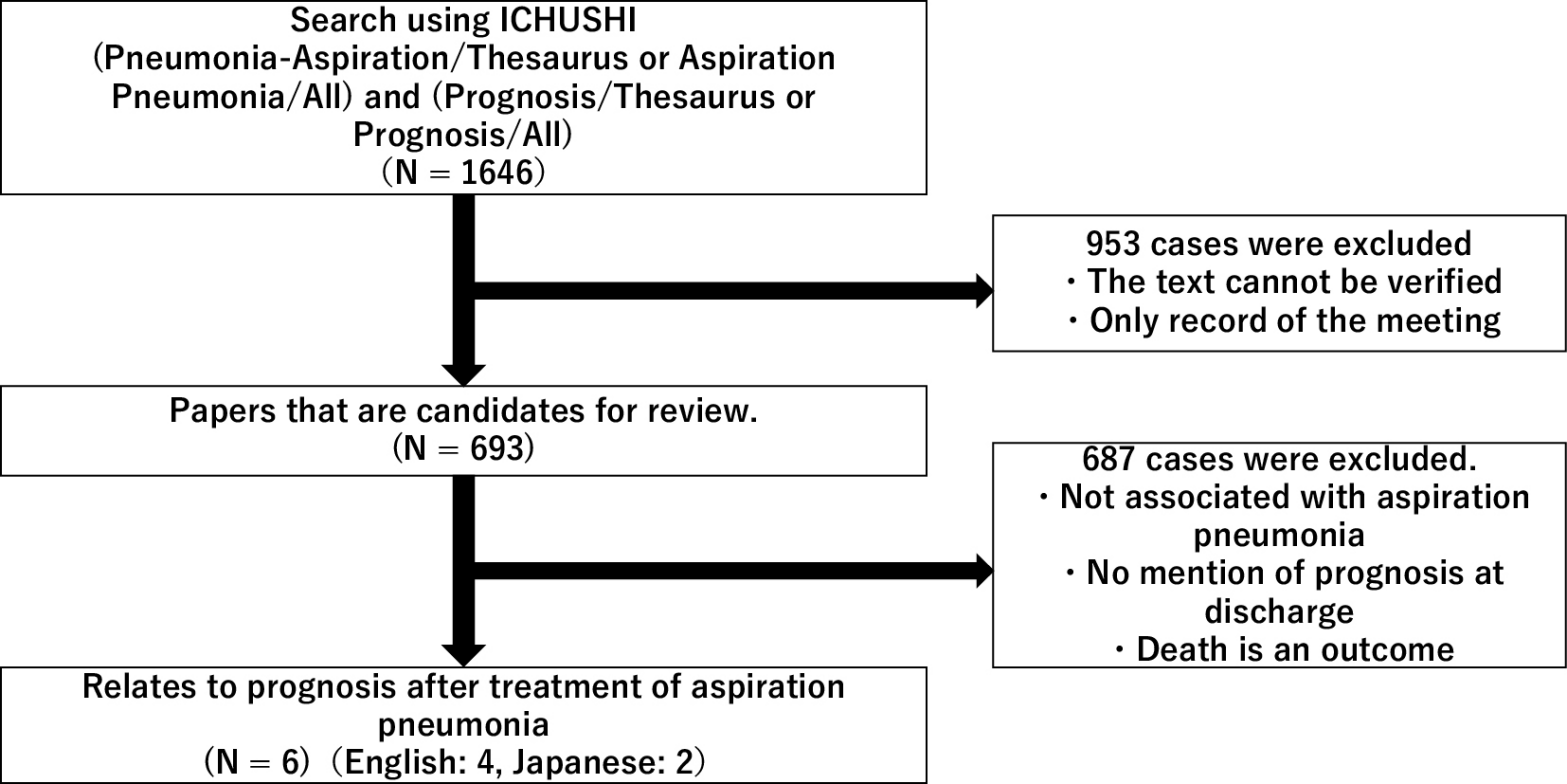
Flow chart of the scoping review.

Data collection was performed by two reviewers. In instances where a discrepancy arose between reviewers, a consensus was reached through mutual consultation. The targeted population for data collection was Japanese patients, and information was retrieved from the Japanese database ICHUSHI (refer to Figure 1). The search strategy employed was defined using the formula “Pneumonia-Aspiration/Thesaurus or Aspiration Pneumonia/All) and (Prognosis/Thesaurus or Prognosis/All.”

The inclusion criteria comprised the involvement of Japanese patients, hospitalization for aspiration pneumonia, and a clearly defined outcome focusing on the availability of oral intake. The exclusion criteria included text unavailability, studies from foreign countries, and cases involving nonhospitalized patients. These criteria were crucial for maintaining the specificity and relevance of the collected data.

Initially, the title and abstract were carefully checked for preliminary screening of relevant papers, and the eligibility of full-text articles was evaluated. Disagreements were resolved through discussion and consensus, and were finally checked. Ultimately, articles for which the full text was inaccessible were eliminated, and the records of these excluded articles were maintained in a supplementary document. These systematic measures ensured a rigorous and targeted approach to data collection, aligning with the study’s objective of identifying risk factors associated with oral intake discontinuation upon discharge in the context of aspiration pneumonia.

#### Risk of bias assessment

The risk of bias for each study was assessed by two reviewers using the Newcastle-Ottawa scale ^(8)^. Each study was classified as low/intermediate/high risk of bias based on the score obtained in three domains: selection, comparability, and outcome. These three domains included eight criteria. Scores ≥7-9, 4-6, and <4 were considered to indicate low, intermediate, and high risk of bias, respectively.

## Results

### Results of the collected papers

A total of 1,646 articles were initially identified using the aforementioned search formula. After excluding conference proceedings and texts with unverified content, 693 articles remained for further consideration (see Figure 1).

The application of the inclusion and exclusion criteria led to the final selection of six articles. These publications, dated between 2015 and 2021, encompassed studies conducted in Japan, by Japanese researchers exclusively focusing on patients hospitalized for aspiration pneumonia (refer to Table 1). The participant demographics revealed a broad age range, with an average age in the 80s. While male dominance was noted in all studies, one study did not report the male-to-female ratio.

**Table 1.** Summary of Studies Included in the Scoping Review.

Authors (Year)	Title	Country	Target population	Number of Patients (M/F)	Average age (min-max)	Language
Tatebe et al. (2021) ^(9)^	Designing a Clinical Pathway Based on Swallowing Functional Assessment and Considering Its Efficacy for Aspiration Pneumonia Inpatients	Japan	Aspiration pneumonia inpatients	94 (43/51)	90 (84-95)	Japanese
Nakamura et al. (2020) ^(10)^	Effect of Early Dysphagia Rehabilitation by Speech-Language-Hearing Therapists in Patients with Severe Aspiration Pneumonia	Japan	Hospitalized patients with severe aspiration pneumonia	226 (138/88)	84 (77-92)	English
Osanai et al. (2020) ^(11)^	Effect of early dysphagia evaluation and dysphagia rehabilitation on patients with aspiration pneumonia	Japan	Aspiration pneumonia inpatients	260 (151/109)	84 (78-89)	Japanese
Ito et al. (2018) ^(12)^	Predictors for achieving oral intake in older patients with aspiration pneumonia: video fluoroscopic evaluation of swallowing function	Japan	Aspiration pneumonia inpatients	160 (99/61)	82 (72-91)	English
Momosaki et al. (2015) ^(13)^	Predictive factors for oral intake after aspiration pneumonia in older adults	Japan	Patients with aspiration pneumonia admitted to an acute care hospital	66611 (37117/29494)	84 (77-91)	English
Iwamoto et al. (2014) ^(14)^	Swallowing rehabilitation with nutrition therapy improves clinical outcomes in patients	Japan	Patients with aspiration pneumonia admitted to an acute care hospital	70 (No data)	73 (61-83)	English

### Analysis items and outcomes

The analysis encompassed various items across studies, including physiological outcomes, for example, age, gender, and body mass index (BMI). Items related to swallowing function, including early rehabilitation, dietary ADL (whether the content is related to daily life or to swallowing), number of days to start swallowing training, mean duration of nonoral intake until evaluation, VF findings, Fujishima dysphagia scale, and FILS (assessing the severity of swallowing ability). Items related to physical activity and daily functioning include Barthel Index (BI) and ADL on evaluation. Disease-related items included A-DROP score, “blood urea nitrogen, saturation of percutaneous oxygen, consciousness disturbance, blood pressure”, nursing and healthcare-associated pneumonia, severe pneumonia, invasive or noninvasive ventilation, items related to underlying disease, “including history of aspiration pneumonia, history of cerebrovascular disease, comorbidities, Charlson Comorbidity Index score, gastrostomy”, items related to nutrition and lifestyle prior to hospitalization, “including place of care before admission, admission from nursing home, moderate or severe nutritional risk, ambulatory before admission, albumin, weight loss before admission, type of hospital admitted, basal energy expenditure (BEE), mid-upper arm muscle circumference, triceps skinfold thickness, and so on” (refer to Table 2).

**Table 2.** Variables of Reviewed Sources.

Authors (Year)	Items compared	Outcome
Tatebe et al. (2021)	BMI, MNA-SF, Barthel Index, place of care before admission, history of aspiration pneumonia, history of cerebrovascular disease	Discharged with oral intake possible/discharged with no oral intake possible or dead.
Nakamura et al. (2020)	A-DROP score, BUN, SpO2, consciousness disturbance, BP, NHCAP, invasive or noninvasive ventilation, comorbidities, moderate or severe nutritional risk, admission from nursing home, ambulatory before admission, FILS score at the start of rehabilitation, early rehabilitation	No alternative nutrition/alternative nutrition
Osanai et al. (2020)	Charlson Comorbidity Index score, place of care prior to admission, dietary ADL Mentor r metropolitan, A-DROP score, Alb, Fujishima Gr, number of days until swallowing training was started	Discharged with oral intake possible/discharged with no oral intake possible
Ito et al. (2018)	Alb, mean duration of nonoral intake until evaluation, ADL on evaluation, VF findings, past history and comorbidities, A-DROP score	Discharged with oral intake possible/discharged with no oral intake possible
Momosaki et al. (2015)	Age, gender, Barthel Index, weight loss before admission, severe pneumonia, A-DROP score, comorbidities, type of hospital admitted, length of stay, gastrostomy	Discharged with oral intake possible/discharged with no oral intake possible
Iwamoto et al. (2014)	BMI, BEE, %AMC, %TSF, support days	Discharged with oral intake possible/discharged with no oral intake possible

BMI: body mass index, MNA-SF: Mini-Nutritional Assessment Short-Form, A-DROP: Age-Dehydration, Respiration, Orientation, Pressure, BUN: blood urea nitrogen, SpO2: saturation of percutaneous oxygen, BP: blood pressure, NHCAP: nursing and health care-associated pneumonia, FILS: Food Intake LEVEL Scale, ADL: activities of daily living, Alb: albumin, Fujishima Gr: Fujishima Grade, VF: swallowing videofluorography, BEE: basal energy expenditure, AMC: mid-upper arm muscle circumference, TSF: triceps skinfold thickness

### Summary of results affecting outcomes

**Figure 2.**
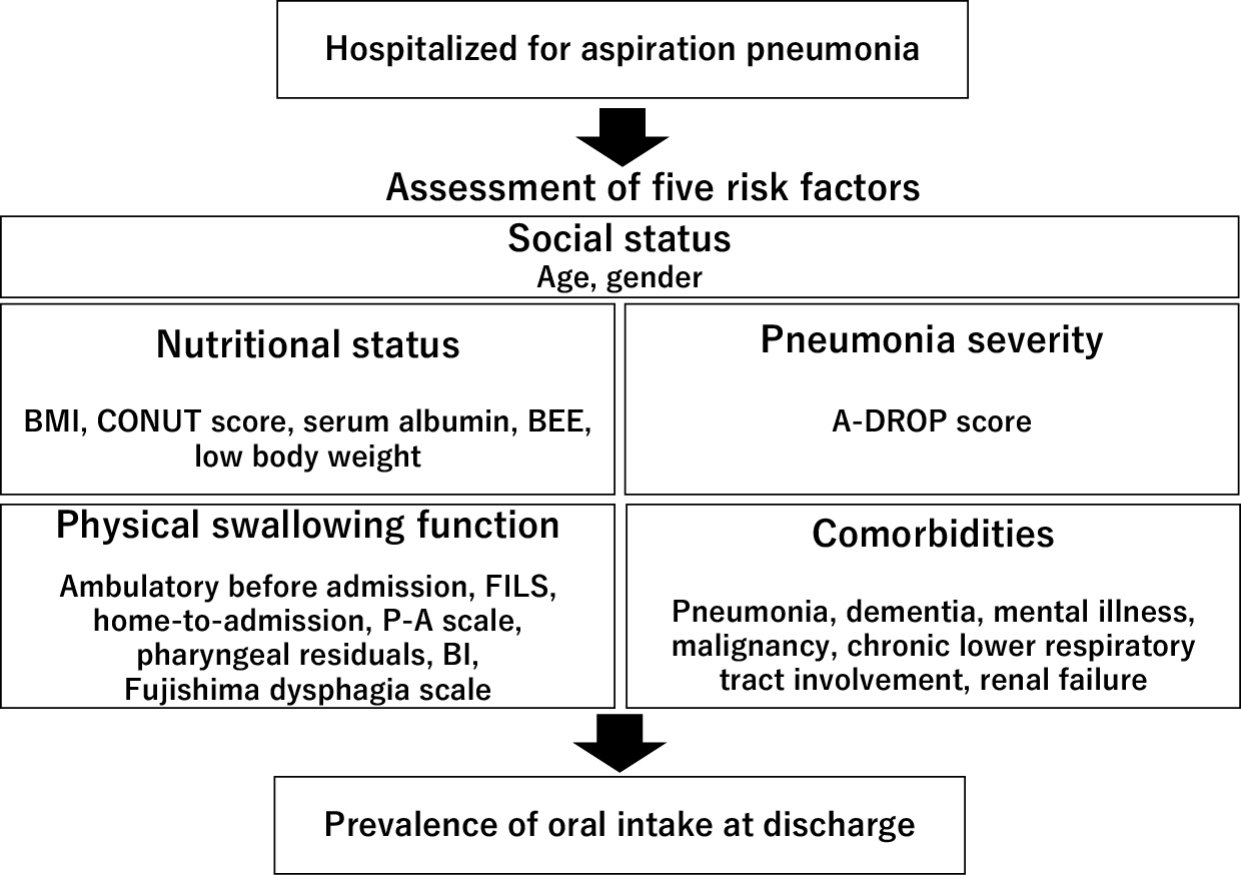
Five risk factors that influence swallowing function.

Each study identified several factors (ranging from a few to around 10) associated with oral intake capability at hospital discharge (refer to Table 3). These factors were broadly categorized into social status, nutritional status, physical swallowing ability, pneumonia severity, and comorbidities (see Figure 2). Nutritional status factors included BMI, Nutritional Status score (CONUT score), albumin, BEE, and low body weight. The factors for physical swallowing function comprised ambulatory ability before admission, FILS score at the start of rehabilitation (average of 5 points in the oral group/3 points in the nonoral group), admission from home and not from other places, bedridden status (not wheelchair or gait), P-A scale (average 2.6 in the oral group/2.3 in the no oral group), presence of residual pharyngeal material, and BI on admission (average 32.5 in the oral group/22.3 points in the no oral group). The severity of pneumonia was assessed using the A-DROP score. Comorbidities included pneumonia, dementia, chronic lower respiratory disease, malignancy, mental illness, and renal failure. Additional factors, such as gender, hospital type, and posthospitalization variables (e.g., early rehabilitation, gastric banding, and length of hospital stay), were also found to be significant.

**Table 3.** Factors Influencing Oral Intake Discharge.

Authors (Year)	Factors leading to oral intake discharge	Factors leading to no oral intake discharge
Tatebe et al. (2021)	Age (89 years), BMI (18.9)	Age (92 years), BMI (17.0)
Nakamura et al. (2020)	CONUT score of 5 or more (63.6 points), ambulatory before admission (49.1%), FILS score at the start of rehabilitation (5), early (5 days or more) rehabilitation (76.3%)	CONUT score of 5 or more (83.0 points), ambulatory before admission (28.3%), FILS score at the start of rehabilitation (3), early (5 days or more) rehabilitation (50.9%)
Osanai et al. (2020)	Hospitalization from home (59.7%), serum albumin (3.1 g/dl), Fujishima Gr (6)	Hospitalization from home (27.8%), serum albumin (2.8 g/dl), Fujishima Gr (2)
Ito et al. (2018)	Serum albumin (3.0 g/dl), mean A-DROP score (2.5), mean FILS score before admission (8.4), mean duration of nonoral intake until evaluation (7.8 days), bedridden ADL (41.3%), mean score of P-A scale (2.6), severity of residual of pharynx, history of pneumonia (26.9%), dementia (56.7%)	Serum albumin (2.7 g/dl), mean A-DROP score (2.2), mean FILS score before admission (7.7), mean duration of nonoral intake until evaluation (11.2 days), bedridden ADL (70.0%), mean score of P-A scale (2.3), severity of residual of pharynx, history of pneumonia (42.9%), dementia (73.2%)
Momosaki et al. (2015)	Women (46.0%), Barthel Index on Admission (32.5), underweight on admission (43.7%), severe pneumonia (like A-DROP score), history of malignancy (8.1%), sepsis (1.7%), cerebrovascular disease (5.5%), oral disease (15.1%), mental disorder (14.9%), chronic lower respiratory disease (8.3%), renal failure (2.7%), academic hospital (4.0%), mean length of stay day (15.3%), gastrostomy in hospital (2.7%)	Women (41.7%), Barthel Index on admission (22.3), underweight on admission (66.9%), severe pneumonia (like A-DROP score), history of malignancy (9.8%), sepsis (3.1%), cerebrovascular disease (5.9%), oral disease (20.0%), mental disorder (16.9%), chronic lower respiratory disease (10.9%), renal failure (3.1%), academic hospital (4.6%), mean length of stay day (63.9%), gastrostomy in hospital (11.8%)
Iwamoto et al. (2014)	BMI (21.8), BEE (1192 kcal)	BMI (19.2), BEE (1067 kcal)

BMI: body mass index, CONUT: controlling nutritional status, FILS: Food Intake LEVEL Scale, ADL: activities of daily living, P-A scale: Penetration-Aspiration Scale

### Risk of bias results

The risk of bias in the six studies was low (Table 4). Possible sources of bias included uncertainty about whether the outcome was present at the start of the study, unclear adjustments for confounding factors, or issues related to the follow-up period.

**Table 4.** New Castle Ottawa Scale for Risk of Bias.

Study	Selection				Comparability		Outcome			Overall
	Represent activeness of the exposed cohort	Selection of the non exposed cohort	Ascertainment of exposure	Outcome not present at start	Age factor	Other factor	Assessment of outcome	Adequate follow-up length	Adequacy of follow up	
Tatebe et al. (2021)	＊	＊	＊		＊	＊	＊	＊		7
Nakamura et al. (2020)	＊	＊	＊	＊	＊	＊	＊	＊	＊	9
Osanai et al. (2020)	＊	＊	＊	＊	＊	＊	＊	＊	＊	9
Ito et al. (2018)	＊	＊	＊	＊	＊	＊	＊	＊	＊	9
Momosaki et al. (2015)	＊	＊	＊	＊	＊	＊	＊	＊	＊	9
Iwamoto et al. (2014)	＊	＊	＊		＊	＊	＊	＊	＊	8

## Discussion

### Implications for practice

The study aimed to identify the risk factors associated with oral intake discontinuation upon discharge in patients hospitalized for aspiration pneumonia. Five factors were identified: social status, nutritional status, pneumonia severity, physical swallowing function, and comorbidities.

#### Social status

Age, a component of the A-DROP score, is likely correlated with increased pneumonia severity and decreased prehospitalization swallowing function. Although gender differences were inconclusive, further studies are warranted.

#### Nutritional status

Factors such as BMI, CONUT score, serum albumin, BEE, and low body weight were associated with poor swallowing function posttreatment. This aligns with existing knowledge linking poor nutritional status with unfavorable rehabilitation outcomes and physical health ^(15)^. The combination of inflammation and undernutrition in pneumonia may contribute to functional decline ^(16)^.

#### Pneumonia severity

Critically ill patients requiring intensive care and oxygen therapy may face challenges in initiating early swallowing rehabilitation due to respiratory instability. Early rehabilitation was found to have an impact on general patients and those with severe pneumonia, highlighting its role in influencing oral intake ^(17), (18)^.

#### Physical swallowing function

Prehospitalization factors such as ambulatory ability, FILS, admission origin, P-A scale, pharyngeal residuals, and BI were identified as influencing outcomes. Poor ADL and swallowing function prior to hospitalization increased the risk of postdischarge difficulties due to disuse ^(19)^.

#### Comorbidities

Both psychiatric and nonpsychiatric disorders were risk factors. Dementia types and mental illness, which affect swallowing function, warrant individualized dietary adjustments. The effects of medication, particularly from antipsychotics, must be considered. Nonpsychiatric disorders, such as a history of pneumonia, malignancy, chronic lower respiratory tract lesions, and kidney abnormalities, were also identified ^(20), (21), (22), (23), (24), (25)^.

Considering the clinical relevance of each factor, future research could explore weighting or categorizing these items for a more accurate predictive model. Additionally, beyond oral intake, the impact of these factors on patients returning to their original place of care and the varied decision-making timelines in clinical practice pose intriguing avenues for further investigation.

### Limitations

One limitation is the focus on inpatients in Japan; however, the generalizability of the findings remains robust when limited to Japan. Discrepancies in participant numbers across studies were observed, but all studies had sufficient sample sizes for analysis, thereby mitigating this concern. Furthermore, speech-language pathologists were involved in the assessment of swallowing function in five of the studies, but in some hospitals in Japan, there is no specialist involved in rehabilitation and dysphagia management. This may affect the resumption of oral intake and the management of patients’ swallowing function; however, it was impossible to summarize who was responsible for the management of dysphagia in each study.

## Conclusion

This study identified five risk factors associated with oral intake discontinuation upon discharge in patients hospitalized for aspiration pneumonia. The evidence generated has practical implications for informing future clinical practice and guiding interventions aimed at optimizing patient outcomes. Further research exploring the nuanced complexities of patient care decisions beyond oral intake is warranted.

## Article Information

### Conflicts of Interest

None

### Author Contributions

KK and ELAMT conceived the research topic. KK contributed to the collection of clinical information, data analysis, and manuscript preparation under the supervision of KSL and ELAMT. All authors critically reviewed and revised the manuscript and approved the final version for submission.

### Approval by Institutional Review Board (IRB)

This systematic review used studies that are published in several medical databases. Ethics approval was not required for this study.
